# Monitoring candidate gene expression variations before, during and after a first major depressive episode in a 51-year-old man

**DOI:** 10.1186/1471-244X-14-73

**Published:** 2014-03-12

**Authors:** Raoul Belzeaux, Jean-Michel Azorin, El Chérif Ibrahim

**Affiliations:** 1 Aix Marseille Université, CNRS, CRN2M UMR 7286, 51 Bd Pierre Dramard, 13344 cedex 15 Marseille, France; 2 APHM, Hôpital Sainte Marguerite, Pôle de Psychiatrie Universitaire Solaris, 13274 cedex 9 Marseille, France; 3 FondaMental, Fondation de Recherche et de Soins en Santé Mentale, Créteil, France

**Keywords:** mRNA, Biomarker, 5HTT, TNF, Antidepressant, Major depression

## Abstract

**Background:**

Although psychiatric disorders are frequently characterized by clinical heterogeneity, high recurrence, and unpredictable prognosis, studies of mRNA expression variations in blood cells from psychiatric patients constitute a promising avenue to establish clinical biomarkers. We report here, to our knowledge, the first genetic monitoring of a major depressive episode (MDE).

**Case presentation:**

The subject is a 51-year-old male, who was healthy at baseline and whose blood mRNA was monitored over 67 weeks for expression variations of 9 candidate genes. At week 20 the subject experienced a mild to moderate unexpected MDE, and oral antidepressant treatment was initiated at week 29. At week 36, the patient recovered from his MDE. After 6 months, antidepressant treatment was discontinued and the subject remained free of depressive symptoms. Genetic monitoring revealed that mRNA expression of *SLC6A4/5HTT* increased with the emergence of a depressive state, which later returned to basal levels after antidepressant treatment and during MDE recovery. *PDLIM5*, *S100A10* and *TNF* mRNA showed also an interesting pattern of expression with regards to MDE evolution.

**Conclusion:**

This case demonstrated the applicability of peripheral mRNA expression as a way to monitor the natural history of MDE.

## Background

In psychiatry, biomarker identification remains at its infancy despite advances in pharmacogenomics [1,2], proteomics, as well as translational sciences [3,4]. In recent years, several investigators have reported variations in mRNA levels between patients and control subjects based on peripheral and accessible tissues and also between pre- and post-treatment conditions in major depressive disorder (MDD) and bipolar disorder (BD) [5-11].

Development of RNA transcripts as biomarkers was facilitated by developments in reverse transcription in combination with quantitative real-time PCR (RT-qPCR), with high sensitivity, specificity, reliability, and at a very affordable costs [12]. Using these methods, we previously tested mRNA expression variations in blood samples from individuals suffering from severe major depressive episode (MDE), with the aim of validating gene candidates proposed by others as biomarkers in major depression [6].

So far, very few studies have validated the potential significance of such biomarkers [5,8,11,13], and longitudinal studies covering a complete depressive episode from symptoms appearance to full remission are still lacking due to difficulty in conducting prospective studies with subjects that have not yet developed depression. Indeed, little is known about natural mRNA variations occurring over more than 6 months, and which mRNAs may correlate to observable clinical features before, during, and after a MDE. Therefore, both long-term longitudinal studies and individual case studies are needed to better understand gene expression variations.

Here, we describe the variation of mRNA expression levels of selected candidate genes associated with mood disorders in a single subject, who was recruited by chance, before, during and after a first MDE. Venous blood was collected from the fasting subject at weeks 0, 2, 8, 29, 41, 43, 50, and 67 in EDTA tubes. Peripheral mononuclear blood cells (PMBCs) were isolated from the blood by Ficoll density centrifugation. Total RNAs were extracted from the PBMCs with the mirVana kit (Ambion) according to the manufacturer’s protocol. The purity and integrity of the RNA was determined by optical density measurements and by the Agilent 2100 Bioanalyzer (Agilent Technologies).

We focused our peripheral gene expression analysis on previously identified putative genes involved in the pathophysiology of major depression and its treatments [6,11,13]. We selected *SLC6A4/5HTT,* a well known serotonin transporter gene associated with depression vulnerability and a target of most antidepressant treatments [14,15], *S100A10,* another potential marker of antidepressant effect encoding p11 [16-18], *PDLIM5*, encoding a protein associated with mood disorders [19,20], and several cytokines/chemokines playing important roles in regulating immune-inflammatory processes (*CCL2*, *IL1B, IL6, IL8, IL10* and *TNF*) [21].

Candidate gene expressions were conducted as previously described [6]. The relative fold change expression (FC) was calculated using the 2^-ΔΔCt^ formula with the DataAssist software (Applied Biosystems, v3.0).

## Case presentation

A 51 year-old male was enrolled as a healthy control in a previously published prospective study of gene expression [6]. Based on the French version of standardized interview validated for healthy control subjects (SCID-NP) [22], the patient did not have a history of psychiatric disorder or other notable medical conditions prior to and at the time of his inclusion. Nevertheless, at week 20 after inclusion, he experienced a mild to moderate MDE, which was diagnosed based on the occurrence of depressive mood, anhedonia, psychomotor retardation, asthenia, and thoughts of death. He consented to stay in the study despite this medical event and was switched to the MDE group of the same study protocol. Following the MDE, the subject retrospectively reported soft depressive signs, such as isolated feelings of worthlessness and hopelessness, that first appeared at week 14 of the study. He initiated oral antidepressant treatment at week 29 (Agomelatine, 25 mg/day) and recovered from his MDE at week 36. After 6 months, the treatment was stopped and the subject remained free of depressive symptoms for the last weeks of our follow-up.

**Figure 1:**
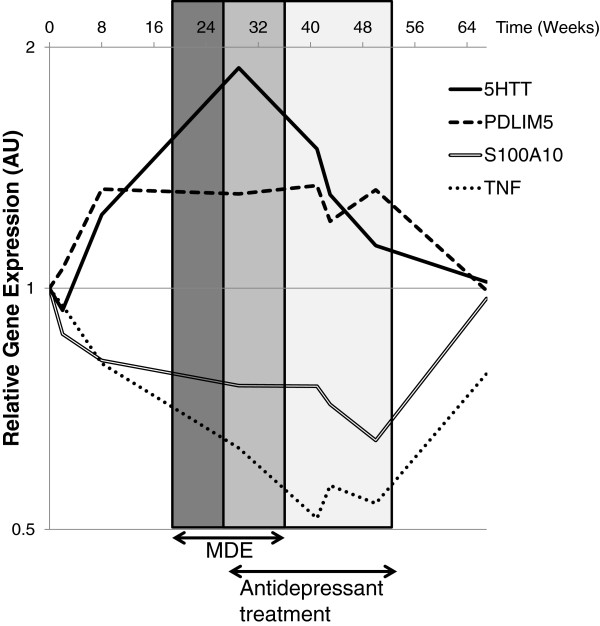
**RT-qPCR analysis of *SLC6A4/5HTT, PDLIM5, S100A10 *and *TNF *mRNA expression in PBMCs collected at weeks 0, 2, 8, 29, 41, 43, 50, and 67.** Patient received antidepressant treatment (Agomelatine, 25 mg/day) between week 29 and week 54. The reference genes used for normalization were: **(i)***RPL38* and *RPLP0* to quantify *S100A10* level of expression, **(ii)***ABL1* and *MAPK1* for *PDLIM5*, **(iii)***SYNJ2* and *ABL1* for *IL8* and *TNF*, **(iv)***SYNJ2*, *SYP*, and *NGFR* for *CCL2*, *IL6*, *IL10* and *SLC6A4/5HTT*. The calibrator sample was sample at week 0. All qPCR experiments were conducted in duplicate and the mean Ct value was used for FC calculation.

Among the genes investigated, the 4 genes (*SLC6A4/5HTT*, *PDLIM5*, *S100A10* and *TNF*) that showed the most interesting variations are shown on Figure 1. The expression of *SLC6A4/5HTT* exhibited a biphasic variation consisting of an increase during the symptomatic phase and a return to normal level after antidepressant treatment. Interestingly, we found a similar pattern of gene expression variations for *PDLIM5*. Of note, *SLC6A4/5HTT* expression was inversely correlated to *IL10* expression (Spearman’s coefficient correlation ρ = −0.71, p = 0.047, false discovery rate (FDR) = 0.161). Conversely, *S100A10* demonstrated variations in the opposite direction compared to *SLC6A4/5HTT*, and was also strongly correlated to *TNF* variation (ρ = 0.86, p = 0.007, FDR = 0.067), *IL1B* (ρ = 0.81, p = 0.015, FDR = 0.067) and *CCL2* (ρ = 0.83, p = 0.010, FDR = 0.067), Finally, *TNF*, which demonstrated an opposite pattern of expression compared to *PDLIM5*, was strongly correlated to important mediators of inflammation such as *IL1B* (ρ = 0.93, p = 0.001, FDR = 0.036), *IL8* (ρ = 0.81, p = 0.015, FDR = 0.067), and *CCL2* (ρ =0.81, p = 0.015, FDR = 0.067).

## Conclusions

We present here, to the best of our knowledge, the first case report of gene expression variations before, during and after an MDE over a large period of 67 weeks. Of note, a previous case report had described a patient suffering from BD with rapid cycling, and evaluated over a period of time the clinical symptoms and gene expression with genome-wide microarray hybridization and RT-qPCR using cells collected from peripheral blood [23]. In that study, genes involved in prostaglandin metabolism and immune system regulation were expressed in an episode-specific manner (i.e., depressed versus manic episodes).

This case report provides two new insights. First, we observed that gene expression variation of selected genes could precede clinical observable manifestations. This observation is of high interest in the context of biomarker identification, particularly for target biomarkers that could be used to track evolution of symptomatology and may be predictive of either relapse or recurrence [13]. The return of *SLC6A4/5HTT* mRNA expression to baseline level after antidepressant treatment could be used as a biological definition and general method to predict remission. Secondly, our results suggest that several biological pathways may play important roles during MDE. This case report indicates that *SLC6A4/5HTT* mRNA regulation could be implicated in MDE independently of treatment effect. Moreover, several genes implicated in immune response were also dysregulated, confirming the immune dysregulation in MDD [24,25].

Our correlation analysis on repeat measurements of gene expression allowed us to propose a potential link between the serotonin pathway and inflammation dysregulation [26,27], as well as a link between *S100A10* and *TNF*, which has been suggested in previous studies [28].

Despite these new insights, the results presented here have some limitations. First of all, it is difficult to draw general conclusions based on an isolated case of MDE patient. Replications are warranted but finding similar cases would require a very large prospective cohort within healthy subjects or individuals at risk for an MDE. Moreover, the inherent physiological or stochastic variations in gene expression could contribute to the observed variations and little is known about variation of gene expression of candidate genes such as *SLC6A4/5HTT*[29]. Finally, we favored a candidate gene approach and restricted our analysis to a few genes of interest. Of note, other candidate biomarker genes such as *FKBP5* could also be informative in such a case and deserve further investigations [30,31].

In conclusion, gene expressions in blood tissue could pave the way to biomarker description in mood disorder as described here. Even if cohort studies are the gold standard to develop such aim, intra-individual variation and single case report could uncover some new insights in the field of personalized medicine.

## Consent

Written informed consent was obtained from the patient for publication of this Case report. A copy of the written consent is available for review by the Editor of this journal.

## Abbreviations

MDE: Major depressive episode; MDD: Major depressive disorder; BD: Bipolar disorder; PBMC: Peripheral blood mononuclear Cell; RT-qPCR: Reverse transcription in combination with quantitative real-time PCR; FC: Fold change; FDR: False discovery rate.

## Competing interests

The authors declare that they have no competing interests.

## Authors’ contributions

RB and EI conceived the study. RB carried out the clinical evaluation. EI performed the genetic experiments. RB and EI analysed the results. All authors drafted, read, and approved the final manuscript.

## Pre-publication history

The pre-publication history for this paper can be accessed here:

http://www.biomedcentral.com/1471-244X/14/73/prepub
